# Disease in Children

**Published:** 1893-06

**Authors:** 


*Disease in Children.* By JAMES CARMICHAEL, M.D. Pp. xvi., 592. Edinburgh: YOUNG J. PENTLAND. 1892.

In a handbook on "Disease in Children" by the lecturer on this important subject in the University of Edinburgh, we naturally look for information at once trustworthy and instructive, and in Dr. Carmichael's book we do not look in vain. It is a handy, compact volume, well arranged, printed in clear type, and thoroughly up to the level of the most recent scientific treatment.

We are pleased to see that early in his work Dr. Carmichael gives a clear statement of the sense—and the only sense—in which the study of children's ailments can be considered a "special" one. He says: "We are dealing with the same diseases as in adult life, modified as they are by the conditions of growth and development going on in the child. It is not strictly correct to talk of diseases *of* children as we would of women, who suffer from ailments peculiarly their own." With this we are quite in agreement.

In the limited space at our disposal, we feel we can hardly do justice to a book evidently the result of much earnest work in the presence of abundant clinical material, but we must point out that the excellent chapters on typhoid fever, tuberculosis, and gastro-intestinal disorders are typical of the manner in which such subjects should be treated in a manual of this kind.

We shall be pleased if Dr. Carmichael can in a future edition see his way clear to give prescriptions in *single* doses, as most busy men dislike the trouble of "calculating back," and much prefer to get at the dose without first having to solve a small arithmetical problem. Altogether, we can recommend this handbook as a safe guide to the subject of which it treats.

